# Rethinking screening mammography in Japan: next-generation breast cancer screening through breast awareness and supplemental ultrasonography

**DOI:** 10.1007/s12282-023-01506-w

**Published:** 2023-10-12

**Authors:** Takayoshi Uematsu

**Affiliations:** Department of Breast Imaging and Breast Intervention Radiology, Shizuoka Cancer Center Hospital, 1007 Shimonagakubo, Nagaizumi, Shizuoka 411-8777 Japan

**Keywords:** Breast cancer screening, Mammography, Ultrasonography, Breast awareness, J-START

## Abstract

Breast cancer mortality has not been reduced in Japan despite more than 20 years of population-based screening mammography. Screening mammography might not be suitable for Japanese women who often have dense breasts, thus decreasing mammography sensitivity because of masking. The J-START study showed that breast ultrasonography increases the sensitivity and the detection rate for early invasive cancers and lowers the rate of interval cancers for Japanese women in their 40 s. Breast awareness and breast cancer survival are directly correlated; however, breast awareness is not widely known in Japan. Next-generation breast cancer screening in Japan should consist of breast awareness campaigns for improving breast cancer literacy and supplemental breast ultrasonography to address the problem of false-negative mammograms attributable to dense breasts.

## Introduction

In many Western countries, screening mammography programs have been established to reduce breast cancer mortality [1–10]. In Japan, population-based screening mammography has been in progress for more than 20 years; however, no reduction in breast cancer mortality has been evident [11, 12]. Screening mammography might, therefore, not be suitable for Japanese women possibly because they often have dense breasts, thus decreasing mammography sensitivity because of masking. Japanese women in their 40 s, a population with a high incidence of breast cancer, are often found to have dense breasts [13].

The Japan Strategic Anti-cancer Randomized Trial (J-START) of supplemental ultrasonography to screen for breast cancer in women 40–49 years of age demonstrated that breast ultrasonography increased the sensitivity and the detection rate for early invasive cancers and lowered the rate of interval cancers in the intervention group compared with the control group [14].

The practice of breast awareness is correlated with breast cancer survival [15], but breast awareness is not widely known in Japan [16].

In this review, the limitations and problems associated with screening mammography in Japan and the keys to the success of Japan’s next-generation breast cancer-screening program—breast awareness and supplemental ultrasonography—are presented.

## Current breast cancer environment in Japan

Of all cancers, female breast cancer had the highest incidence, with 2.3 million new cases (representing 11.7% of all cancer cases worldwide) in 2020 [7]. Breast cancer is the fifth leading cause of cancer-related mortality, with 685,000 deaths. Breast cancer incidence rates, which had been historically low, are rising rapidly East Asia, associated with dramatic lifestyle changes such as decreased reproduction, increased obesity, and physical inactivity [7].

Breast cancer is the highest incidence malignancy in Japanese women, with approximately 94,000 new cases having been diagnosed in 2019. One of nine women in Japan will develop breast cancer in her lifetime, and breast cancer is expected to continue as the fourth most common cause of cancer-related death in Japanese women, with approximately 15,000 deaths having been recorded in 2020. Furthermore, the breast cancer mortality rate in Japan has been increasing [11], and the disease tends to occur in younger women: its incidence is the highest for women in their 40 s [13].

## History and current status of breast cancer screening in Japan

The Japan Breast Cancer Screening Program, which is held every 2 years, started in 2000 for women 50 years of age and older with mediolateral oblique one-view mammograms. Since 2004, the program has included women in their 40 s and has used two-view mammograms. This unique program combines this recommended screening mammography with clinical breast examination despite clinical breast examination not being recommended since 2016. The program also has no upper age limit; however, since 2021, 40–69-year-old Japanese women have been actively recommended to undergo breast cancer screening. Two types of opportunistic screening programs are also available: one is a population-based screening program offered by local governments, and the other is a premium service provided by companies. Some women pay the total out-of-pocket cost for breast cancer screening using NINGEN DOCK (private complete medical checkup) [13]. Double reading and comparison reading of mammograms are recommended in Japan.

## No breast cancer mortality reduction from screening mammography in Japan

In many developed Western countries, breast cancer mortality rates have been declining since the early 1990s because of the implementation of early breast cancer detection programs, especially screening mammography in the 1980s [1]. Routine population-based screening mammography is the only screening method that has been demonstrated to reduce breast cancer mortality. Early detection through mammography can identify cases of small, nonpalpable, less advanced breast cancers, which are associated with lighter treatment and higher survival rates. Japan has had organized population-based screening mammography for more than 20 years; however, as already mentioned, mortality rates in Japan are still increasing [11, 12].

A recent study that used a simulation model to assess the relative contributions of screening and treatment to the abatement of breast cancer mortality in the United States reported that the estimated overall reduction in the breast cancer mortality rate was 49% in 2012. Of that reduction, 37% was associated with screening mammography, and 63% was associated with advances in treatment [17].

Japan is a well-resourced and well-developed country. Almost all Japanese women with breast cancer can receive state-of-the-art chemotherapy and hormone therapy in real time because the Japanese public medical insurance systems bear 70% of the treatment cost; patients pay only 30%. In addition, Japan has a high-cost medical care benefits system that compensates for excessive medical care expenses. Japanese women with breast cancer thus already benefit from advances in breast cancer treatment because cost is less of a burden.

As an author, I, therefore, hypothesize that the breast cancer mortality rate is increasing in Japan because of a lack of benefit from screening mammography.

## Recipe for the success of screening mammography: high sensitivity and high participation rates

Population-based screening mammography has revealed causal relationships between breast cancer mortality and two main parameters: the sensitivity of the test and the participation rate [18]. Breast radiologists can improve the sensitivity of their imaging techniques; however, participation rates are a more complex issue.

**Fig. 1:**
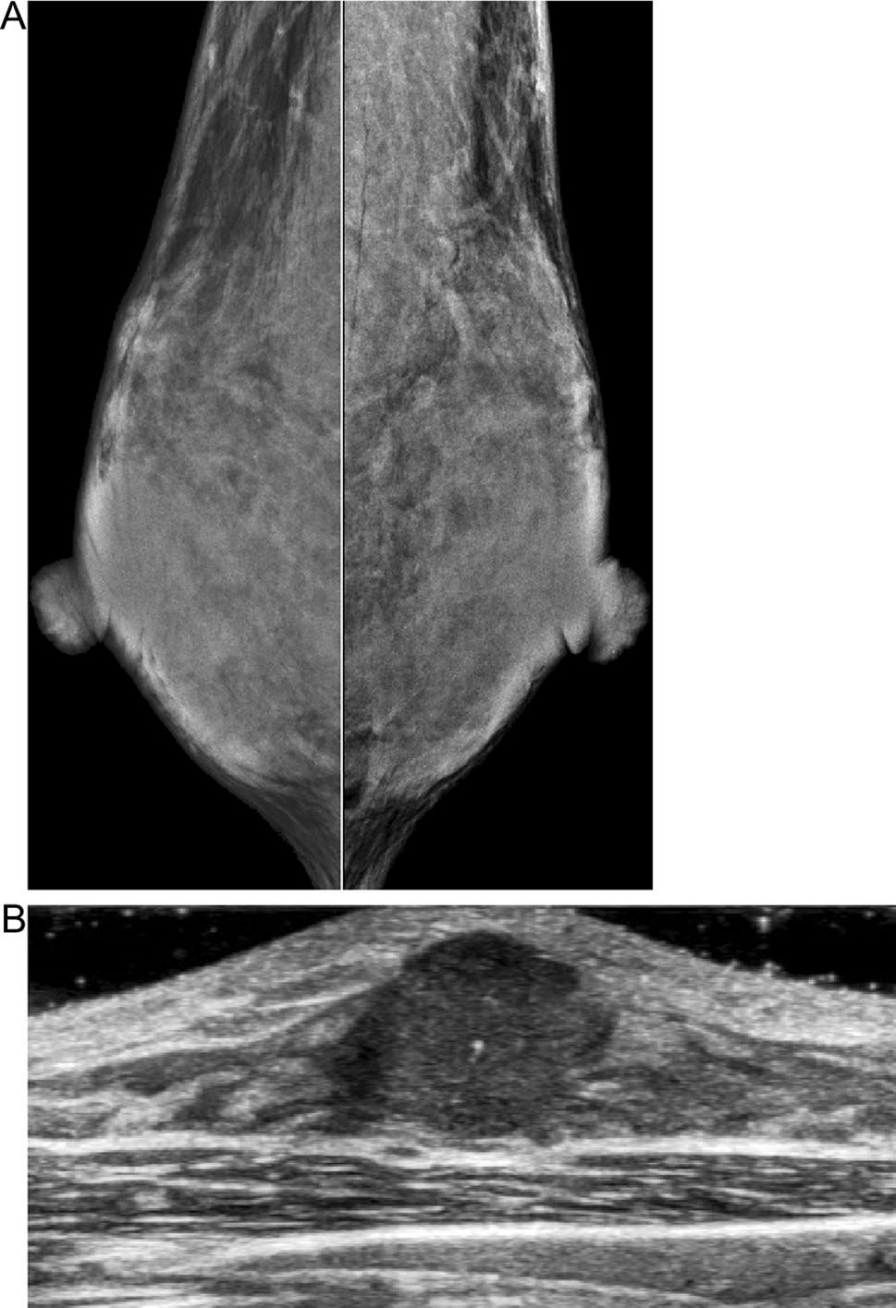
Mammographically occult invasive ductal carcinoma measuring 19 mm was detected by ultrasonography in a 47-year-old woman with extremely dense breasts. **A** There are no mammographic abnormalities due to the masking effect. **B** The gray-scale breast ultrasonography image shows a hypoechoic mass with microlobulated margins

Mammographic sensitivity is affected by masking—that is, cancers that cannot be detected because of the superimposition of overlapping radiopaque dense breast tissue on an underlying malignancy when a three-dimensional breast is imaged in a two-dimensional mammogram (Fig. 1). A decrease in mammography sensitivity with increasing breast density has been established [19, 20].

Compared with Australian women, Japanese women are 15 times more likely to have high breast density. Approximately, 90% of Japanese women 40–49 years of age have heterogenous and extremely dense breasts; only 38.5% of Australian women 40–49 have breasts with those characteristics [21]. In Japan, the age-specific incidence of female breast cancer peaks during the 40 s [13]; for Western women, the peak occurs in the 60–69 age group.

The sensitivity of mammography alone in truly asymptomatic Japanese women in their 40 s was reported to be 47.4% [22]. However, that focus on truly asymptomatic women affected only the numerator of the sensitivity calculation, not the denominator. The sensitivity might, therefore, have been slightly underestimated. However, that potential underestimation of the sensitivity (47.4%) is likely small, because the 38% (45/117) of cancers detected in the control group of J-START were palpable by clinical breast examination; that is, the tumors were larger, and more easily detectable on mammograms, which increased the sensitivity (77.0%) of screening mammography. Breast cancer-screening programs should provide mammography only to asymptomatic women with nonpalpable tumors [23].

## Which is more important to breast cancer screening, the sensitivity or the participation rate?

The incidence of and mortality from breast cancer have been steadily increasing in Japan and are expected to continue rise in Korea as well [24]. To mitigate the increases, a > 70% participation rate and > 70% sensitivity for screening mammography are desirable [18]. The participation rate is approaching 70.0% in Korea [25]. However, the mortality rate in that country has still been increasing, suggesting that, for breast cancer mortality reduction, the sensitivity of mammography screening is more important than the participation rate.

In Japan, the participation in screening mammography is only 47% [26], and that rate might be an overestimate, because it was determined based not on data from a national breast cancer-screening database, but from the results of a comprehensive survey of living conditions in Japan. The results of the comprehensive survey of living conditions always include errors related to the respondents’ lapse of memory [26].

## Paradigm shift to supplemental ultrasonography-based breast cancer screening

Japanese women, particularly those in their 40 s, seem to benefit little from screening mammography alone. More-effective breast cancer-screening modalities are needed. Supplemental screening modalities—including ultrasonography, digital breast tomosynthesis and breast magnetic resonance imaging—have been proposed to increase the sensitivity and the detection rates for early-stage breast cancer in women with dense breasts. However, no global consensus to recommend the use of supplemental breast cancer screening modalities in such women has been reached [13].

Breast ultrasonography has been proposed as a possible supplemental modality in breast cancer screening given mammography’s low sensitivity related to masking [13, 27]. Ultrasonography is an inexpensive, convenient, readily available, and radiation-free breast imaging modality that also avoids the need for breast compression. Furthermore, a meta-analysis comparing mammography alone with supplemental screening ultrasonography reported an approximately 40% increase in the cancer detection rate for women with dense breasts [28]. In addition, J-START, the world’s first large-scale, randomized controlled trial of supplemental screening ultrasonography in women 40–49 years of age, demonstrated that supplemental ultrasonography not only increased the sensitivity for and the detection rate of early invasive cancers in the intervention group compared with the control group, but also lowered the rate of interval cancers [14, 29]. Although the mortality rate is the most important parameter for evaluating the efficacy of supplemental screening ultrasonography, preliminary results from J-START are essential contributors to the implementation of next-generation breast cancer-screening programs in Japan. Given the high breast cancer incidence in women in their 40 s in Japan, any mortality benefit probably reflects the high sensitivity of supplemental of supplemental screening ultrasonography in women with dense breasts.

Unlike other supplemental modalities, ultrasonography is highly operator-dependent and thus could lead to many false-positive results for women with dense breasts. Its positive predictive value might, therefore, be lower and its specificity limited. Quality control will be particularly important to help minimize screening-associated harms. In addition, achieving familiarity with breast ultrasonography techniques based on histopathologic anatomic knowledge will be critical in detecting subtle abnormal lesions such as ductal carcinoma in situ [30, 31].

## Future direction of next-generation breast cancer screening in Japan

High throughput makes ultrasonography the most realistic supplemental modality for a population-based breast cancer-screening program in Japan. However, the modality is highly operator-dependent, requiring real-time adjustments to gain, dynamic range, contrast, depth of field, and positioning of the examiner and patient. False-positive results and biopsy rates have been higher in women undergoing supplemental ultrasonography [14, 32], raising the possibility that costs might substantially increase, as estimated in the United States [33]. Quality control is, therefore, critical, particularly in the screening setting. Quality assurance guidelines, including quality control guidelines for breast screening ultrasonography systems themselves; education and training programs for operators; and interpretation criteria are all needed. J-START has prepared such guidelines [34, 35]. An overall assessment system for intensive breast cancer screening using mammography and adjunctive ultrasonography has also been developed in Japan [36], as have quality assurance and quality control guidelines for J-START [37].

However, the effectiveness and feasibility of ultrasound-based breast cancer screening in Japan remain uncertain; a critical dependency would have to be met before implementation. Technologists certified to acquire ultrasound images and doctors certified to read those images are in short supply. According to 2022 data from the Japan Central Organization of Quality Assurance of Breast Cancer Screening, the number of technologists certified for breast ultrasonography screening is 25% of the number of technologists certified for mammography screening, and the number of doctors certified to read ultrasonography images is 23% of the number of doctors certified to read screening mammograms (Table 1).

**Table 1:** Number of certified doctors and technologists involved in breast cancer screening in Japan

	A	B	C	D	Total
Certificated doctors for mammography screening	3723	6947	3189	1514	15,373
Certificated doctors for breast ultrasonography screening	1058	1479	967	94	3598
Certificated technologists for mammography screening	9071	3906	3099	2025	18,101
Certificated technologists for breast ultrasonography screening	1971	1639	918	23	4551

A, B, C, and D mean certificate-ranks for breast cancer screening. People ranked A or B can engage in breast cancer screening individually. People ranked C or D can engage in breast cancer screening under the supervision of people ranked A or B

The automated breast ultrasound system (ABUS) is a well-designed screening tool because of the separation of image acquisition and interpretation. The system has been investigated as a solution to some of the drawbacks of handheld ultrasonography, such as operator dependency, time consumption, lack of reproducibility and standardization [38]. The use of ABUS might, therefore, be one of the solutions for ultrasound-based breast cancer screening in Japan, especially given the limited availability of technologists certified for screening ultrasonography. Given the current state of screening mammography in Japan, an honest discussion about implementing such an adjunctive screening program should be conducted.

## Breast awareness

Breast awareness is a practice that has been advocated in the United Kingdom since the early 1990s and that is now accepted as one measure that is mitigating breast cancer death worldwide [39, 40]. Breast awareness and breast cancer survival have been proved to be associated [15].

In Japan, breast awareness is now a recognized part of breast health promotion and education. It was introduced in October 2022, together with the breast awareness four-point code (Table 2), and has replaced breast self-examination (BSE) in the guidance from Japan’s Ministry of Health, Labour and Welfare.

**Table 2:** Breast awareness four-point code in Japan

1. Know what is normal for your breasts
2. Look and feel for any changes in your breasts
3. Go to a doctor without delay
4. Attend routine screening if you are aged 40 or over

Breast awareness and BSE are not the same. BSE is a regular, repetitive palpation using a rigorous set method that must be properly performed by women at the same time each month. Several meta-analyses of randomized trials reached the conclusion that teaching BSE has no effect on breast cancer mortality and that it has potential harms, including psychological harm and unnecessary imaging tests and biopsies in women without cancer. The U.S. Preventive Services Task Force breast cancer-screening guidelines recommend against teaching BSE, having issued the practice a grade D (more harm than benefit) [39, 41]. With breast awareness, women no longer have to worry about examining their breasts in a particular way or at a particular time. Instead, breast awareness simply asks that they familiarize themselves with their breasts as a normal part of caring for their bodies [39]. Breast awareness encourages women to understand what is normal for them by viewing and feeling their breasts. Most women will know how their breasts look and feel simply through daily activities such as washing and dressing, although this knowledge might be subconscious [39]. Thus, breast awareness is not about searching for cancers; it is about forming a lifestyle habit of caring for one’s breasts almost unconsciously. It is, therefore, crucial that breast awareness and BSE should be distinguished during education sessions.

In Japan, approximately, 70% of breast cancers are detected by women themselves [42]. High cancer symptom awareness has been associated with good cancer survival even when tumors are palpable [15]. Improving the recognition of early symptoms and encouraging prompt visits to the doctor for those symptoms is important. Breast awareness should also help to detect interval cancers, which might be missed because of false-negative mammograms attributable to the masking effect in women in their 40 s with dense breasts. Increasing the practice of breast awareness in Japanese women will thus decrease the frequency of advanced breast cancer. Currently, however, breast awareness is not widely known in Japan, with a recognition rate of only 5% in Japanese women [16].

## Risk-stratified breast cancer screening in Japan

Risk-stratified breast cancer screening could optimize the benefits of screening while minimizing the harms and has become a frequent discussion topic in recent years [43, 44]. Risk-stratified breast cancer-screening programs will be a key strategy in next-generation breast cancer screening. J-START might be considered to have been an intensive, risk-stratified breast cancer-screening program. A secondary analysis of the J-START data revealed that the contribution of adjunctive ultrasonography did not depend on breast density (Table 3) [14, 29] and that the age-specific breast cancer incidence is highest for Japanese women 45–49 years of age [13]. As already discussed, mammographic breast density is greater in Japanese women in their 40 s than in older women, and high breast density is also a robust and independent predictor of breast cancer risk in Japanese women [13]. Ultrasonography could thus be a critical modality for detecting invasive cancers in intensive risk-stratified breast cancer-screening programs, especially for women in their 40 s who are at elevated risk.

**Table 3:** Screening performance of J-START among women in their 40 s from the secondary analysis

	Intervention group	Control group	*P* value
Dense breasts			
Number	5797	5593	
Number of cancers (%)	41 (0.7)	24 (0.43)	0.04
Number of interval cancers	3	10	0.04
Sensitivity	93.2	70.6	< 0.001
Specificity	85.4	91.7	< 0.001
Non-dense breasts			
Number	3908	3915	
Number of cancers (%)	27 (0.7)	14 (0.36)	0.04
Number of interval cancers	2	9	0.03
Sensitivity	93.1	60.9	< 0.001
Specificity	89	91.9	< 0.001

Intervention means screening mammography + ultrasonography + clinical breast examination. Control means screening mammography + clinical breast examination. Reference: (29)

Screening mammography effectively detects early cancers; however, its effectiveness depends on the test sensitivity and the participation rate [18]. Recently, it became clear that participation rates can be increased by deployment of risk-stratified screening programs [44–46]. Studies to explore the effects of such programs are needed.

## Conclusion

Breast cancer mortality reduction crucially depends on the sensitivity of breast cancer screening. Typically, Japanese women have dense breasts that require screening beyond mammography. Screening ultrasonography is the most realistic modality for such women, especially those in their 40 s. The practice of breast awareness is known to be associated with breast cancer survival; however, breast awareness is not widely known in Japan. The next-generation approach to breast cancer screening in Japan should consist of breast awareness campaigns and the introduction of supplemental screening ultrasonography, with subsequent studies to assess whether a reduction in breast cancer mortality is achieved after this risk-stratified breast cancer-screening program is adopted.

## Data Availability

Data sharing is not applicable to this article as no new data were created or analyzed in this study.

## Abbreviations

J-START: The Japan Strategic Anti-cancer Randomized Trial
BSE: Breast self-examination
ABUS: Automated breast ultrasound system
